# Erratum: BatTool: an R package with GUI for assessing the effect of white-nose syndrome and other take events on Myotis spp. of bats

**DOI:** 10.1186/s13029-014-0028-9

**Published:** 2014-12-20

**Authors:** Richard A Erickson, Wayne E Thogmartin, Jennifer A Szymanski

**Affiliations:** Upper Midwest Environmental, Science Center, U.S. Geological, Survey, 2630 Fanta Reed Road, 54603 La Crosse, WI USA; Division of Endangered Species, U.S. Fish and Wildlife Service, 555 Lester Avenue, 54650 Onalaksa, WI USA

## Erratum

After publication of this work [1], we were notified that part of the graphical user interface failed to function correctly. Specifically, the white-nose syndrome take scenarios did not load correctly. The command line aspects of the package were unaffected. This error has been corrected and new code attached.

## Additional file

Additional file 1
**This file contains the needed files to run the model as well as the package in both tar.gz format for Linux and OS X as well as the .zip _le for Windows.**
