# Supplementary figures and images for: BatTool: an R package with GUI for assessing the effect of White-nose syndrome and other take events on Myotis spp. of bats
Source: Source Code Biol Med. 2014 May 6;9:9. doi: 10.1186/1751-0473-9-9 (PMC4050442; doi:10.1186/1751-0473-9-9)

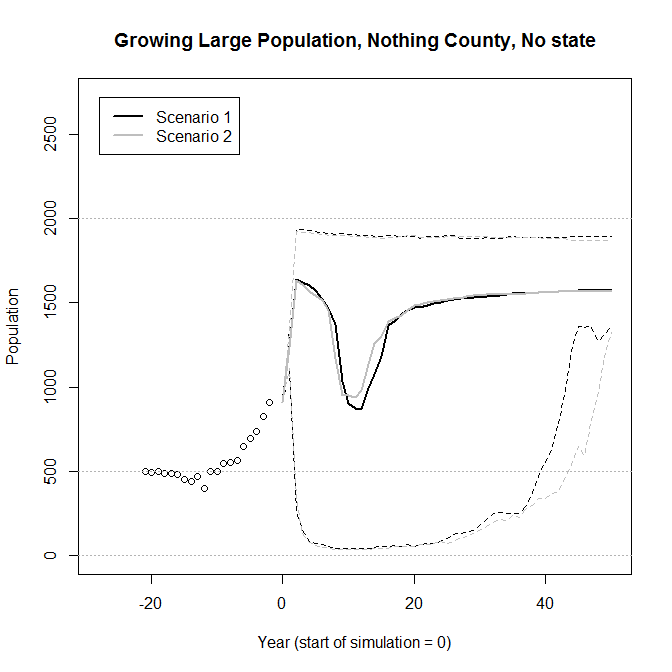

Supplement: Additional file 2 — This file contains the readme file for instillation, the csv files that belong in the working directory, and the complied Windows R package as a ZIP file. This file is all Windows users will need to install and run the package. [file 1751-0473-9-9-S2.zip › BatTool_files/ResultsSingleHib/case/PopulationTrendGraph.tiff]

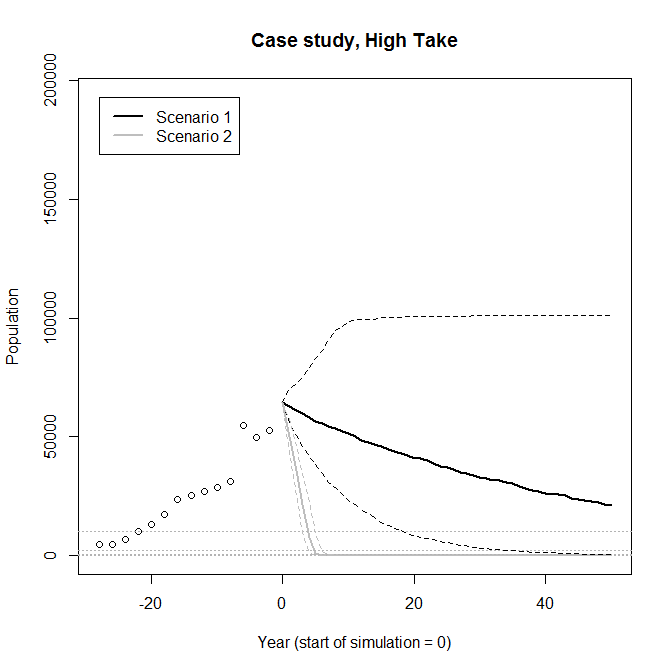

Supplement: Additional file 2 — This file contains the readme file for instillation, the csv files that belong in the working directory, and the complied Windows R package as a ZIP file. This file is all Windows users will need to install and run the package. [file 1751-0473-9-9-S2.zip › BatTool_files/ResultsSingleHib/CaseStudyHigh/PopulationTrendGraph.tiff]
