# Supplementary material for: BatTool: an R package with GUI for assessing the effect of White-nose syndrome and other take events on Myotis spp. of bats
Source: Source Code Biol Med. 2014 May 6;9:9. doi: 10.1186/1751-0473-9-9 (PMC4050442; doi:10.1186/1751-0473-9-9)
Supplement: Additional file 1 — This file contains the raw BatTool code. [file 1751-0473-9-9-S1.zip › BatTool_5.0.tar!/BatTool/inst/doc/GUI_User_Manual.pdf]

Single Hibernaculum Matrix Projection Tool GUI: Instructions and Explanations

Contents

1 Important notes and tips for the GUI **1**

2 Installing BatTool **2**
2.1 Step-by-step install directions 2
2.2 Using the Tool 3

3 Required file for GUI **3**

4 GUI walkthrough **3**
4.1 Hibernacula selection 4
4.2 Lambda 4
4.3 Critical Parameter 95% Intervals 4
4.4 Female Take 5
4.5 WNS 5
4.6 Other parameters 5
4.7 Graphing and results options 6
4.8 Simulation Options 6
4.9 To run 6

5 Results **6**

`BatTool` is a package for people seeking to understand the effects of WNS or other stressors on *Myotis* bats. This document describes how to use the GUI component of the package. The document is a basic introduction on using the GUI and additional documentation may be found in the `readme.txt` file as well as the *Source codes for Biology and Medicine* manuscript (Link URL here once published). To access or leanr more about the command line interface for `BatTool`, load the package, and then open the package help file:

```
> library(BatTool)
> ??BatTool
```

1 Important notes and tips for the GUI

- Any white box can be edited by the user. Hit ENTER for your changes to be recorded.
- Any grey triangle can be expanded or contracted by clicking on it. If all triangles are expanded, the RUN button may not be viewable depending up your screen setting.
- Two different scenarios are compared using this tool.

2 Installing BatTool

The first time you use BatTool, you will need to install the package. Subsequent times, you will only need to load the package, unless you have installed a new version of R. You will need to change the working directory in R for both installation and use of the package. The working directory should contain the following user files:

- `IndianaBatAndHibData.csv`
- `LambdaEstimatseFromObservations.csv`
- `WNS_other_1.csv`
- `WNS_other_2.csv`
- `whitenoseBeginYears.csv`
- `whitenoseProbabilitiesIb.csv`
- `whitenoseProbabilitiesLbb.csv`
- `take parameters.csv`

2.1 Step-by-step install directions

1. Open R and change your working directory to the location of package (BatTool_X.X.zip) where X.X is the version number (e.g., 5.0). This should also be the location where you want to use the package (e.g., a file in ”My documents/MyBatAssessment”). This may be done through either the R's menu system or through terminal commands in R.

   - With R's menu system:
     File -> Change dir
     and then navigate and select your file.
     There are video tutorials of this step on web. As an example a Google search for
     “change working directory r windows”
     found a video tutorial demonstrating this process:
     `http://www.youtube.com/watch?v=CCMVaRRLqYw`
   - Through the terminal in **R**:

     ```
     getwd() ##Current working directory
     setwd("c:/My Documents/BatProject/Assessment")
     ##Make sure to keep everything in this folder
     ##NOTE THE BACKSLASHES INSTEAD OF FORWARD SLASHES
     getwd() ##Notice the change in working directory
     ```

     The file path (“c:/My Documents/BatProject/Assessment”) will depend you computers setup. Also, please not the backslashes instead of the forward slashes.

2. Install the required package `gWidgetstcltk` with either the **R** GUI (Toolbar and menu system):

   - The **R** GUI:
     Packages -> Install Package(s)
   - **R** command:
     install.packages(”gWidgetstcltk”)

3. Install the BatTool package with either the *R* GUI (Toolbar and menu system):

- The R GUI:
  Packages -> Install Package(s) from local zip files...
- The R command line:
  install.packages("./BatTool_X.X.zip", repo = NULL)
  Where X.X is the version number (e.g., 5.0)

This installation step will need to be repeated if either a new version of R is installed on your computer or a new version of the tool is released and you wish to upgrade.

2.2 Using the Tool

1. Change your working directory to the place where you are keeping the required files (See Step 1 in Section 2.1 for directions).
2. Load the package:
   library(BatTool)
3. Launch the GUI:
   demo("GUIcode")
4. Make sure your working directory also contains the necessary files (See Section 3)

3 Required file for GUI

A set of files are included in the `User.zip` or `UserFWS.zip` file. The FWS edition contains hibernacula locations that are not approved for public release. These files include:

- `whitenoseBeginYear.csv`
  Year white-nose syndrome occurred or is expected to occur based upon work by Frick et al.
- `whitenoseProbabilitiesIb.csv`
  FWS-derived consequences of WNS on Indiana bats. This file contains the estimated minimum and maximum effect on survival. The file also contains an effect of birth rate.
- `whitenoseProbabilitiesLBB.csv`
  Frick et al consequences of WNS on Little Brown bats. This file contains the estimated minimum and maximum effect on survival. The file also contains an effect of birth rate.
- `WNS_other_1.csv`
  User specified consequences of WNS for scenario 1.
- `WNS_other_2.csv`
  User specified consequences of WNS for scenario 2.
- `LambdaEstimatesFromObservations.csv`
  Modeled estimates of population rate of change.
- `IndianaBatAndHibData.csv`
  Population counts for wintering populations.

4 GUI walkthrough

This section contains a walkthough of the different sections of the GUI. Each subsection roughly corresponds to a grey triangle box area.

4.1 Hibernacula selection

This is the header of the GUI. Enter the hibernacula ID number in the upper left hand corner white box. Once entered, the name, county, state and recovery unit number will appear as the window title. If you don't know the ID number, click on the button on the left entitled "Hibernaculum number: (Click to lookup in R)." A scrollable window inside R will open up that lists the Hibernaculum names and numbers. This window does not need to be closed to continue.

4.2 Lambda

This group begins expanded. NA The mean level is outside of the parentheses, and an approximate 70% confidence interval is inside the parentheses. The default option is at the complex level, but the hibernaculum and recovery unit level are also included an options. If there is not enough data at the hibernaculum or complex levels, only NA will be an option at that level. Selecting a value from the drop down menu will populate the 'Lower:' and 'Upper:' white boxes, all of which are editable. The lambdas drawn during the simulation will be a draw with uniform probability between the lower and upper limits.

4.3 Critical Parameter 95% Intervals

Critical Parameter 95% Intervals: The number in brackets in the upper left corner indicates the number of combinations of the 12 listed parameters that achieve a lambda within the lower and upper limits of lambda as entered in the above boxes. Each listed parameter then shows an upper and lower limit of a 95% interval that will be used in the simulation. The listed parameter acronyms stand for as follows:

JWS: Juvenile winter survival

AWS: Adult winter survival

NSS: Non-breeder summer survival

JSS: Juvenile summer survival

ASS: Crude index of sexual attraction

PFS: Pup fall survival

JFS: Juvenile fall survival

AFS: Adult fall survival

JP: Juvenile propensity to breed

AP: Adult propensity to breed

JB: Juvenile breeding success

AB: Adult breeding success

Users may not edit these values.

4.4 Female Take

In all cases, the first box, 'Take:', is the number of females to be removed from the population with the beginning year in the second box, 'Begin Year'. The third box, 'Duration', is the number of years that the take will persist. It should be noted that only females are modeled within the simulation, but the total population (males and females) is reported in the output. The population of females is then multiplied by two at the end of each year of each simulation to correspond to total population, under the assumption that there are equal numbers of each sex. The total take will be apportioned in the model in accordance with the adult to juvenile proportion for that year (i.e. if 100 are taken in the winter, and the adult proportion is 0.81, 81 will be taken from the adult population and 19 taken from the juvenile proportion for that winter). Seasonal take important because of when reproduction occurs as well as seasonal mortality. The same take, but occurring in different seasons will produce different population trajectories. To examine this effect, compare the same take (e.g., 4 bats beginning in year 1 for 10 years), but occurring during different seasons.

4.5 WNS

For each scenario, the survival probabilities post-WNS have upper and lower bounds. These bounds can be selected for each scenario with the drop-down menu. There are 4 options within the drop-down menu:

1. If 'No WNS' is selected, there will be no WNS mortality, and all survival probability bounds are 1.

2. If 'LB ests' is selected, for the first seven years, each year has a separate beta-distribution that defines the survival probability. These are based on the little brown bat estimates produced by Frick et al. Survival probability is NOT a uniform draw between the lower and upper limits for the first seven years. For years 8 and beyond, the model is a uniform draw between the upper and lower bounds (i.e. if the upper bound is 0.95 and the lower bound is 0.9, any number between 0.9 and 0.95 is equally likely for the survival probability). The default for years 8+ is complete survival. These estimates are based upn work by Frick et al.

3. The 'IB ests' are from expert opinions solicited by the USFWS.

4. The "other" tables are included in case the user does want to edit the original files.

The data structure of the 'IB est', 'LB ests', and 'Other' csv files are the same, but contain different vales. ALL SURVIVAL PARAMETERS ARE EDITABLE. The parameter may either be edited in the GUI or the CSV files. If you want to see and/or change the survival and breeding reduction probabilities (the breeding reduction probabilities default to a negligible reduction of 0.001 for each year), click on the 'Scenario 1' or 'Scenario 2' buttons. A 'Data Editor' window will open inside R, every element of which can be edited Your edits will be saved once you 'X-out' the window with the red X button in the upper right hand corner. You MUST close this window in order to proceed with the tool, whether or not you have changed it.

Given that a hibernaculum is uninfected, its yearly probability of infection is set to 0.096. This is editable by the user. If a hibernaculum is already infected or you know (somehow) what year it will become infected, enter the year as years after the start of the simulation (e.g., if your data ends in 2010 and you think WNS will arrive in 2015, enter 5, not 2015). NA

4.6 Other parameters

The starting adult proportion is 0.8, which is an adult:juvenile ratio of 4:1. Environmental stochasticity is set to 0.04. This means that the 12 parameters have a random number uniformly drawn from between -0.04 and +0.04 added to each parameter, independently from each other and for each

simulation run. An internal (i.e., non-user) function ensures parameter values stay within the a biologically realistic range (e.g., 0 to 1 for survival). Demographic stochasticity is the default, which treats each births and deaths as a binomial probability within the model. Without demographic stochasticity, each parameter is simply a multiplier upon the population. For example, under demographic stochasticity, if AWS=0.95 on a population of 10 bats, the number of bats surviving the winter will be 9.5 on average, but will vary randomly around that number, given binomial variation (e.g., five draws might look like {9, 8, 9, 10, 6}). Under no demographic stochasticity, the number of bats surviving the winter will always be exactly 9.5.

4.7 Graphing and results options

A plot is produced for each simulation run, and the color of the lines can be entered here. You may choose the first scenarios color in the 'Color:' drop down menu, and the second scenario is automatically chosen as the lighter of the chosen color. If you want to overlay multiple scenarios on the same plot, check the 'Add to plot' box. If you want to add credible intervals, check the 'Add _ % credible intervals' box (defaults to 95%). The graph produced will have a title, which is editable in the 'Graph/Results title:' white box.

4.8 Simulation Options

The default number of years to simulate is 50, and the default for the number of simulations to run is 1000 replicates (i.e., each simulation uses a different set of parameter values and stochastic variables). These are editable, and the optimum number of simulations to run will vary and will be mostly dependent upon the width of the lambda interval.

4.9 To run

Click on the 'RUN'; button. The results will be placed in a folder inside the 'ResultsSingleHib' folder inside your working directory. NA The default name is 'temp' To restore default values to ALL of the boxes, click on 'Restore defaults.' Once the run button is clicked, an updating status bar should display below the run button, indicating what simulation number is currently being run.

5 Results

Graphs produced after a run have a solid dark line for the median of the projections for scenario 1, and a lighter solid line for the median of the scenario 2 projections. Dashed lines of the same color indicate credible intervals. NA A copy of this graph is provided automatically in the results folder, 'Population trend graph.tif', but it may also be right clicked on and copied.

A summary of the simulation parameter settings and results are saved within the user specified "Results folder". The `Results summary` file includes the model settings and probabilities of reaching different thresholds within a giving time period. The `YearlyResultsScenario_X.txt` (where X is the scenario, either 1 or 2) contains the yearly population sizes. The `ParameterValueSceanrio_X.txt` file contains the parameter values for scenario X. The `Scenario X whitenose survival probabilites.txt` contains the WNS survival probabilities used for scenario X. The `Scenarios_X_take_parameters` contains the take parameters used for a given scenario.
