# Supplementary material for: BatTool: an R package with GUI for assessing the effect of White-nose syndrome and other take events on Myotis spp. of bats
Source: Source Code Biol Med. 2014 May 6;9:9. doi: 10.1186/1751-0473-9-9 (PMC4050442; doi:10.1186/1751-0473-9-9)
Supplement: Additional file 2 — This file contains the readme file for instillation, the csv files that belong in the working directory, and the complied Windows R package as a ZIP file. This file is all Windows users will need to install and run the package. [file 1751-0473-9-9-S2.zip › BatTool_files/BatTool_5.0.zip!/BatTool/html/00Index.html]

R: Bat Population Model for Indiana and Little Brown Bat

# Bat Population Model for Indiana and Little Brown Bat

---

## Documentation for package ‘BatTool’ version 5.0

- DESCRIPTION file.
- User guides, package vignettes and other documentation.
- Code demos. Use demo() to run them.

## Help Pages

|  |  |
| --- | --- |
| BatTool-package | Bat Population model for Indiana Bat and Little Brown Bat |
| BatTool | Bat Population model for Indiana Bat and Little Brown Bat |
| envStoch | Environmental stochasticity adder |
| lambdaSampler | Samples lambda lookup table |
| lkup | Lookup Table of Parameter for the Population Model |
| main\_pop | Main population model |
| pop\_stochastic | Stochastic population model |
