# Supplementary material for: BatTool: an R package with GUI for assessing the effect of White-nose syndrome and other take events on Myotis spp. of bats
Source: Source Code Biol Med. 2014 May 6;9:9. doi: 10.1186/1751-0473-9-9 (PMC4050442; doi:10.1186/1751-0473-9-9)
Supplement: Additional file 2 — This file contains the readme file for instillation, the csv files that belong in the working directory, and the complied Windows R package as a ZIP file. This file is all Windows users will need to install and run the package. [file 1751-0473-9-9-S2.zip › BatTool_files/BatTool_5.0.zip!/BatTool/doc/index.html]

R: Vignettes and other documentation

# Vignettes and other documentation

---

## Vignettes from package 'BatTool'

|  |  |  |  |  |  |
| --- | --- | --- | --- | --- | --- |
| BatTool::GUI\_User\_Manual |  | Introduction to BatTool GUI | PDF | source | R code |
