# Supplementary material for: Erratum: BatTool: an R package with GUI for assessing the effect of white-nose syndrome and other take events on Myotis spp. of bats
Source: Source Code Biol Med. 2014 Dec 20;9:122. doi: 10.1186/s13029-014-0028-9 (PMC4289230; doi:10.1186/s13029-014-0028-9)
Supplement: Additional file 1 — This file contains the needed files to run the model as well as the package in both tar.gz format for Linux and OS X as well as the .zip _le for Windows. [file 13029_2014_28_MOESM1_ESM.zip › ESM/1181704568151516_add7.pdf]

Erickson *et al. Source Code for Biology and Medicine* 2014, **9**:9
http://www.scfbm.org/content/9/1/9

SOURCE CODE FOR
BIOLOGY AND MEDICINE

RESEARCH Open Access

BatTool: an R package with GUI for assessing the effect of White-nose syndrome and other take events on *Myotis* spp. of bats

Richard A Erickson[1,2*], Wayne E Thogmartin[1] and Jennifer A Szymanski[3]

Abstract

Background: *Myotis* species of bats such as the Indiana Bat and Little Brown Bat are facing population declines because of White-nose syndrome (WNS). These species also face threats from anthropogenic activities such as wind energy development. Population models may be used to provide insights into threats facing these species. We developed a population model, `BatTool`, as an **R** package to help decision makers and natural resource managers examine factors influencing the dynamics of these species. The **R** package includes two components: 1) a deterministic and stochastic model that are accessible from the command line and 2) a graphical user interface (GUI).

Results: `BatTool` is an **R** package allowing natural resource managers and decision makers to understand *Myotis* spp. population dynamics. Through the use of a GUI, the model allows users to understand how WNS and other take events may affect the population.
The results are saved both graphically and as data files. Additionally, **R**-savvy users may access the population functions through the command line and reuse the code as part of future research. This **R** package could also be used as part of a population dynamics or wildlife management course.

Conclusions: `BatTool` provides access to a *Myotis* spp. population model. This tool can help natural resource managers and decision makers with the Endangered Species Act deliberations for these species and with issuing take permits as part of regulatory decision making. The tool is available online as part of this publication.

Keywords: Conservation biology, Natural resource management, Endangered species, Wildlife ecology, Population dynamics

Background

Bats in the *Myotis* genus, including the Little Brown Bat (*Myotis lucifugus*) and Indiana Bat (*M. sodalis*), face population-level threats in the eastern United States and Canada. The emerging fungal disease White-nose syndrome (WNS) has caused massive decreases in population sizes and is predicted to contribute to further declines as the disease spreads farther west across North America [1]. The Little Brown Bat was one of the most common bat species in the eastern United States until the arrival of White-nose syndrome. The drastic decrease in Little Brown Bat populations has led the U.S. Fish and Wildlife Service to consider listing the species under the Endangered Species Act [2]. Conversely, the Indiana Bat was one of the first species listed under the Endangered Species Act [3]. In addition to WNS, these two species face other threats from anthropogenic activities such as wind energy development [4,5].

Population models have emerged as one method to understand and manage wildlife populations in light of uncertainty [6]. These models may include biologically important attributes such as different life stages (e.g., juveniles and adults). Decision makers and resource managers use these models to explore different scenarios. Possible scenarios might include no management (status quo) or different management approaches. Possible stressors that might be included within the models include harvest (e.g., hunting or fishing) or other takes such as energy development or habitat loss. These models may also address variability and uncertainty through the inclusion of stochasticity. Models may include variability relating to

*Correspondence: rerickson@usgs.gov
[1]Upper Midwest Environmental Science Center, U.S. Geological Survey, 2630 Fanta Reed Road, 54603 La Crosse, WI, USA
[2]Cherokee Nation Businesses, 165 South Union Blvd Suite 700, 80228 Lakewood, CO, USA
Full list of author information is available at the end of the article

© 2014 Erickson et al.; licensee BioMed Central Ltd. This is an Open Access article distributed under the terms of the Creative Commons Attribution License (http://creativecommons.org/licenses/by/2.0), which permits unrestricted use, distribution, and reproduction in any medium, provided the original work is properly credited. The Creative Commons Public Domain Dedication waiver (http://creativecommons.org/publicdomain/zero/1.0/) applies to the data made available in this article, unless otherwise stated.

small population sizes (demographic stochasticity), variability associated with environmental conditions (e.g., droughts vs wet years; environmental stochasticity), and uncertainty in parameter estimates (e.g., 2 births and 1 death per year vs 10 births and 9 deaths per year) [7].

Thogmartin et al. [8] developed a population model for studing the effects of WNS on *Myotis* spp. The original model was written in Matlab (MATLAB and Statistics Toolbox Release 2012b, The MathWorks, Inc., Massachusetts, United States), but the source code was not included as part of the publication nor easily usable by decision makers at agencies such as the U.S. Fish and Wildlife Service. We developed this model into an **R** [9] package to assist decision makers in using the code. **R** was chosen because it is Open Source and freely available to interested users. The model we present within this manuscript contains two different components: 1) a command-line deterministic and stochastic model and 2) a graphical user interface (GUI). The command line option allows **R**-savvy users to include the model as part of their own script. The GUI was specifically developed for U.S. Fish and Wildlife Service decision makers desiring a tool specifically implementing the model presented by Thogmartin et al. [8].

Methods

Underlying population model

Thogmartin et al. [8] previously published the population model forming the backbone of `BatTool`. We include a flow chart of the model (Figure 1), the equations (Equations 1,2,3,4,5,6,7,8 and 9), and variables (Table 1) within this article as well as an overview of the biology underlying the model. Additional analysis of the model was published with the original article [8].

Indiana Bats and Little Brown Bats migrate between summer maternity roost sites and winter hibernacula. Pups are born at roost sites and then migrate to hibernacula during the fall. At this point, the pups become first-year breeders (colloquially referred to as juveniles within our model). The juveniles overwinter at the hibernacula. The juveniles then migrate to summer roost sites during the spring. Our model does not directly consider spring migration mortality. A proportion of the juveniles breed. The breeding and non-breeding juveniles may have different survival rates within the model for the summer and fall seasons. The juveniles migrate back to hibernacula during the fall and become adults. The adults then overwinter and migrate in the spring to the summer roost sites. Like the juveniles, there are both breeding and non-breeding adults. After summer, the adults migrate to the hibernacula during fall. This cycle continues until the bats die [4,5].

Our model reports the bat population size during winter that would be found at a specific hibernacula. This was done because most bat surveys are conducted at the hibernacula and the winter populations are best understood and monitored for both the Little Brown Bat and Indiana Bat. Our model only follows females within the model. The input population is divided by two and the results are multiplied by two under the assumption of an even gender distribution. This is a common assumption in population ecology because males do not limit population size in many non-monogamous species including bats [4,5,7]. Our model is a matrix model (a series of discrete-time difference equations) that follows the population P through time. $P(t)$ is a two-entry vector with the top entry being the number of juveniles and the bottom entry being the number of adults at time t, where t is the time in year. The projection matrix, A, moves the population forward one year (Table 1):

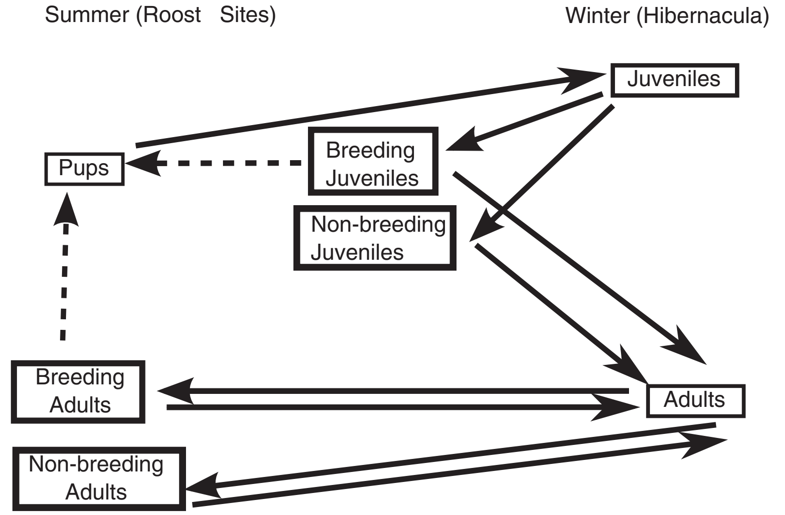

Figure 1 Conceptual map of population model. Solid lines indicate life stage changes and dashed lines indicate births.

$$\mathbf{A} = \begin{pmatrix} 0.5 \times \mathrm{JWS} \times \mathrm{PJ} \times \mathrm{JSS} \times \mathrm{bJ} \times \mathrm{PFS} & 0.5 \times \mathrm{AWS} \times \mathrm{PA} \times \mathrm{ASS} \times \mathrm{bA} \times \mathrm{PFS} \\ \mathrm{JWS} \times \mathrm{pJ} \times \mathrm{JSS} \times \mathrm{JFS} + \mathrm{JWS}(1-\mathrm{pJ})\mathrm{NSF} & \mathrm{AWS} \times \mathrm{pA} \times \mathrm{ASS} \times \mathrm{AFS} + \mathrm{AWS}(1-\mathrm{pA})\mathrm{NSF} \end{pmatrix}. \quad (1)$$

The population at the next year is

$$P(t+1) = \mathbf{A}P(t). \quad (2)$$

We decomposed the projection matrix $\mathbf{A}$ (Equation 1) into the seasonal projection matrices in order to facilitate seasonal "take" and include WNS mortality during the winter. Although take is formally defined under the Endangered Species Act of 1973 to include "harass, harm, pursue, hunt, shoot, wound, kill trap, capture, or collect, or to attempt to engage in any such conduct", our model considers all take as mortality-causing events.

$$\mathbf{A} = (\mathbf{A}_{sfNR} + (\mathbf{A}_{faR} \otimes \mathbf{A}_{suR}))\mathbf{A}_{sp}\mathbf{A}_{wi} \quad (3)$$

where $\otimes$ is the outer product (element-wise matrix multiplication function). $\mathbf{A}$ is decomposed into 5 matrices (Equations 4,5,6 and 7. The spring and fall projection matrix for non-reproducing individuals becomes

$$\mathbf{A}_{sfNR} = \begin{pmatrix} 0 & 0 \\ (1-\mathrm{pJ})\mathrm{FJS} & (1-\mathrm{pA})\mathrm{FAS} \end{pmatrix}. \quad (4)$$

The summer projection matrix for reproducing individuals becomes

$$\mathbf{A}_{suR} = \begin{pmatrix} \mathrm{pJ} \times \mathrm{JWS} \times 0.5 \times \mathrm{bJ} & \mathrm{pA} \times \mathrm{AWS} \times 0.5 \times \mathrm{bA} \\ \mathrm{pJ} \times \mathrm{JWS} & \mathrm{pA} \times \mathrm{AWS} \end{pmatrix}. \quad (5)$$

Table 1 Model parameters names and symbols

Parameter symbol	Parameter name
JWS	Juvenile winter survival
AWS	Adult winter survival
NSS	Non-reproducing juvenile and adult summer and fall survival
JSS	Reproducing juvenile summer survival
ASS	Reproducing adult summer survival
PFS	Pup fall survival
JFS	Juvenile fall survival
AFS	Adult fall survival
JP	Proportion of juveniles reproducing
AP	Proportion of adults reproducing
JB	Births per juvenile
AB	Births per adult
A0	Starting adult population
J0	Starting juvenile population
nYears	Number of years to run mode
K	Carrying capacity of population
wnsSur	WNS survival

The spring projection matrix becomes

$$\mathbf{A}_{sp} = \begin{pmatrix} 1 & 0 \\ 0 & 1 \end{pmatrix}. \quad (6)$$

The winter projection matrix becomes

$$\mathbf{A}_{wi} = \begin{pmatrix} JWS & 0 \\ 0 & AWS \end{pmatrix} \mathrm{wnsSur}. \quad (7)$$

This allows the seasonal take parameters (winter τ_{wi}; spring τ_{sp}; summer τ_{su}; and fall τ_{fa}) to be inserted into the projection matrix:

$$P(t+1) = (((\mathbf{A}_{sfNR} + (\mathbf{A}_{sfNR} \otimes \mathbf{A}_{suR}))(\mathbf{A}_{sp}(\mathbf{A}_{wi}P(t) - \tau_{wi}) - \tau_{sp}) - \tau_{su}) - \tau_{fa}). \quad (8)$$

A simple, ceiling carrying capacity, K, is also used within the model. Once K is reached, $\mathbf{A}$ becomes the identity matrix. The value for K can either be specified by the user or come from population survey data. `BatTool` also includes optional stochasticity. Environmental stochasticity is included by modifying the input parameter with a uniform distribution, *parameter* $\pm$ Uniform(-envs, envs), where "envs" is a user-specified value. A safeguard is also included to ensure the parameter stays within (0, 1). Demographic stochasticity may also be included within the model. When demographic stochasticity is included, a binomial distribution replaces the simple matrix calculations. As an example, the number of juveniles surviving winter would become

$$P_J(t+1) = \mathrm{Binomial}(P_J(t), \mathrm{JWS} \times \mathrm{wnsSur}). \quad (9)$$

The births are also replaced by a binomial distribution. This is appropriate because each female *Myotis* bat may only produce a maximum of 1 offspring per year. Another distribution would be needed if an individual could produce more than one offspring (e.g., Poisson).

Data inputs

`BatTool` includes several different data inputs (Table 2). The `Lambda table` is incorporated within the package because this table is not changed by the user and the file is large. Including the file as an `.Rda` file decreased file size and decreased package load time. The other tables are placed in the working directory where the user may modify values found within the table.

Lambda table

The ratio of the population at year $t+1$ compared to the year t is commonly called lambda in population ecology

Table 2 Model input tables, file names, and location for the Little Brown Bat (LBB) and Indiana Bat (IB)

Table name	File name	File location
Lambda table	`lkup`	`Package data`
Lambda estimates	`LambdaEstimatesFromObservations.csv`	Working directory
Hibernacula table	`IndianaBatAndHibData.csv`	Working directory
LBB WNS infection table	`whitenoseProbabilitiesLBB.csv`	Working directory
IB WN infection table	`whitenoseProbabilitiesIB.csv`	Working directory
WNS infection arrivals	`whitenoseBeginYears.csv`	Working directory

[10]. This is because the growth rate for a linear model (such as our matrix projection model) is also the eigen value, which is commonly represented with the Greek letter lambda (λ) [11]. The annual population growth rates for the Indiana Bat and Little Brown Bat may be estimated from regular hibernacula sampling. The exact parameter values for our population model are unknown due to a paucity of data for *Myotis* spp. However, threats such as WNS and wind energy development may have direct impacts on specific parameters. The theoretical minimum lambda value is 0 and corresponds to all individuals dying off in one year. The theoretical maximum lambda value is 1.5 and corresponds to all individuals living and each female (half of the population) producing 1 offspring. The `lambdaSampler` function within our package returns a set of parameter values from this table for a given range of lambda values. The `lambdaTable` describes uncertainty associated with lambda values. The lambda table is also used with the `LambdaEstimatestable`.

LambdaEstimates table

The `LambdaEstimates` table contains estimated lambda values for each mentioned hibernaculum. This table is populated with hibernaculum-specific population rates of change [8].

Hibernacula table

The `Hibernacula` table lists hibernacula names, counties, take values, and observed population counts. The hibernacula counts are plotted as part of the output. The carrying capacity, K, defaults to be 1.5 $\times$ the maximum observed population at a hibernacula. Also, the starting population within the model is the last year of the observed population counts, but this value can be changed in the GUI by the user. The take description includes the

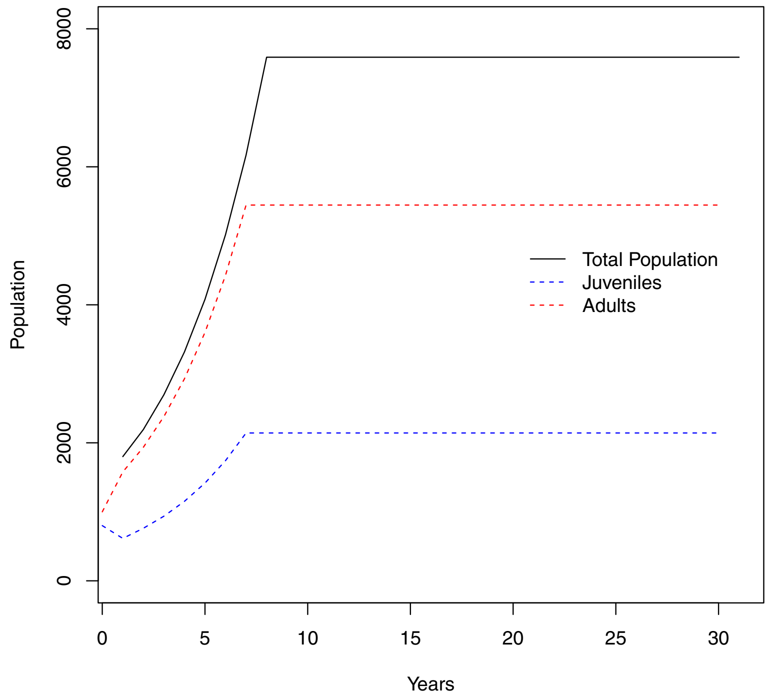

Figure 2 Example deterministic model output from the `main_pop` function.

start, duration, and amount occurred in each season. We included an example table that the user can amend in their own studies.

WNS Infection tables

The `WNSInfection` Probability Table describes species-specific patterns in the decrease in overwinter survival caused by WNS. There are two tables: one for the Indiana Bat and another for the Little Brown Bat. Both tables contain a minimum and maximum value for probability of survival. This value is the minimum and maximum survival during the winter after WNS arrival (e.g., 0.2 implies only 20% of bats survive the disease). A uniform distribution is used to sample this range and a different value is used within each simulation year of the stochastic model. Different survival rates are applied for up to 20 years after WNS exposure. The table also allows amendments to both adult and juvenile birth rate potentially caused by WNS exposure. The model currently assumes that there will be resistance developed following WNS arrival [8]. This resistance could result from an evolutionary, physiological, or behavioral change. The Little Brown Bat survival estimates are based upon work reported by Frick et al. [1] whereas the Indiana Bat estimates are from expert opinions elicited by the U.S. Fish and Wildlife Service. An alternative table reflecting user opinion may also be used with the GUI by selecting "Other Scenario 1" or the "Other Scenario 2" drop down box and reading in a `.csv` file titled either `other_scenario_1.csv` or `other_scenario_2.csv` placed in the working directory.

WNS Infection arrival date table

This table contains the predicted time of arrival for WNS arriving in different locations in the eastern United States. The GUI uses this information to model the arrival of WNS. This table, as with all other model parameters in `BatTool`, are modifiable by the user to reflect study-specific hypotheses.

Results and discussion

Package installation

This package may be installed by downloading it from the journal's additional materials. We have included both the raw package ending in `tar.gz` (Additional file 1) and a file compiled for Windows ending in `.zip` (nested within Additional file 2). Additionally, File 2 is a zip file that also contains data necessary for the GUI to Run. To install the package, use the package installer included as part of **R** (see `?install.packages` for help). Additional install directions are included as part of the of the `readme.txt` file located in the Additional file 2. The `gWidgetstcltk` package and required dependencies are needed for the GUI to work. After installing the

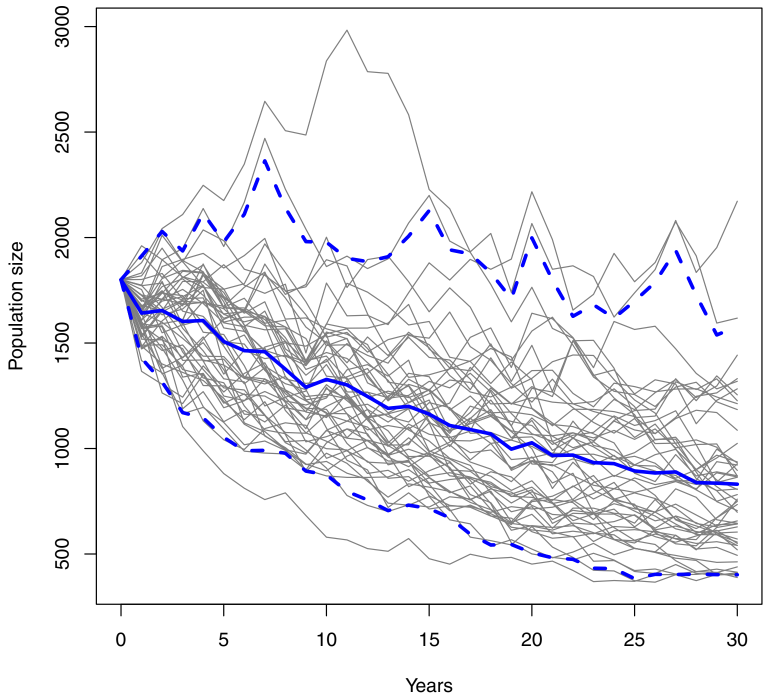

Figure 3 Example of the stochastic model output from the `pop_stochastic` function. This includes 50 stochastic simulation runs as well as the model's mean (solid blue line) and 95% confidence interval (dashed blue lines).

package, use the `library(BatTool)` to load the tool.

Command lines tools

The two major functions within the package are the deterministic model (`main_pop`) and the stochastic model (`pop_stochastic`). To see an example of the deterministic model, use the following lines of code:

```
> library(BatTool)
> example(main_pop)
```

This will produce Figure 2. In this example, the population grows until it reaches its carrying capacity. The example also shows the juvenile and adult populations.

The stochastic model runs multiple simulations and includes several different options worth noting. Running the example for the function will show 50 example population trajectories with the mean and 95% credibility interval overlayed on the plot (Figure 3). This function requires that the number of simulations (or replicates) be specified by the user. Three levels of stochasticity may be run with this model (Figure 4). The model includes parameter uncertainty for any lambda value or range of lambda values. Environmental stochasticity may be specified with a value of zero indicating no environmental stochasticity. Demographic stochasticity may be turned on. Both types of stochasticity may be included. The different levels of stochasticity are also shown with the following example for this function.

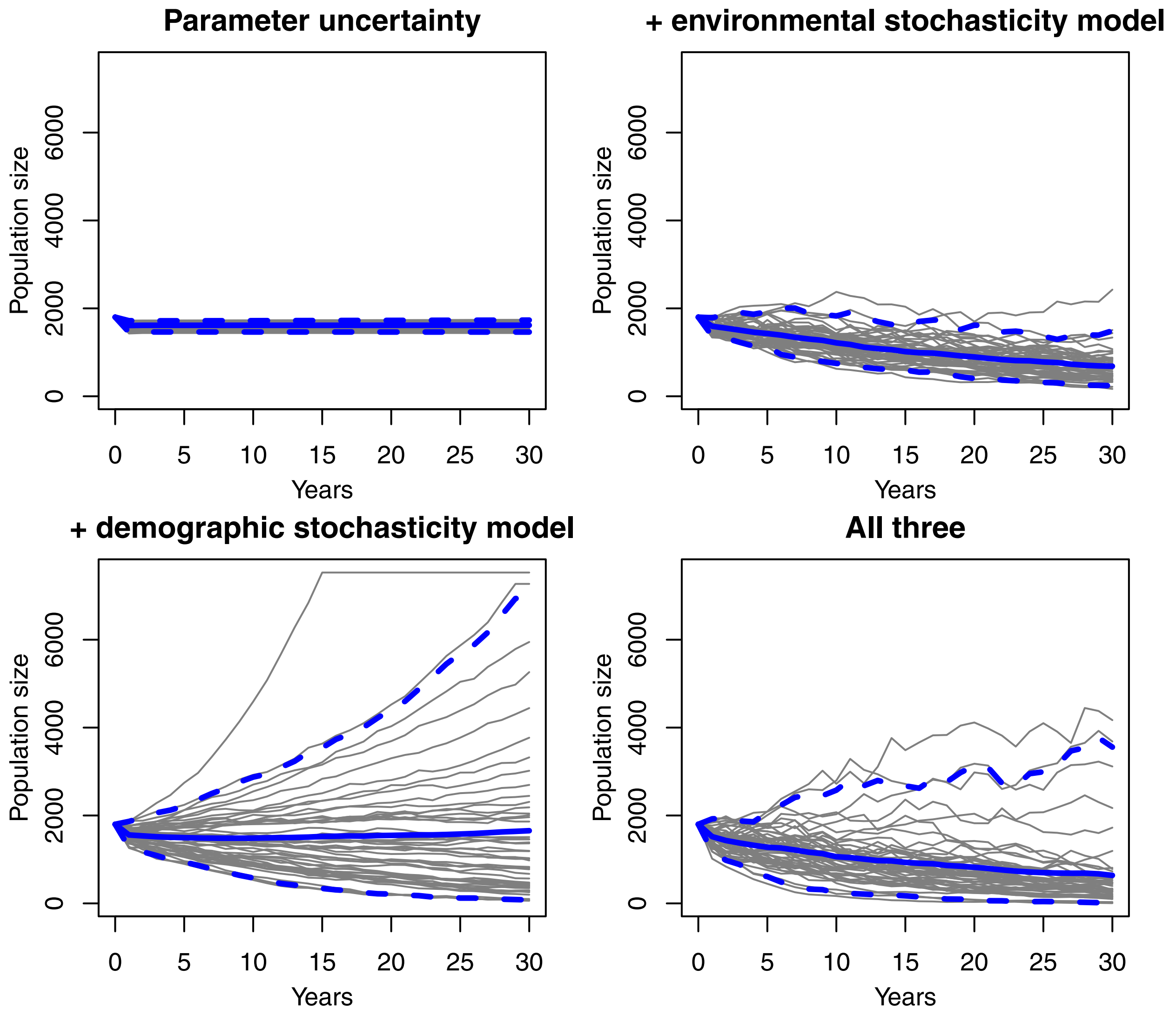

Figure 4 Examples of different types of stochasticity. The black lines are individual replicates, the solid blue line is the mean output, and the dashed blue lines are the models 95% confidence interval.

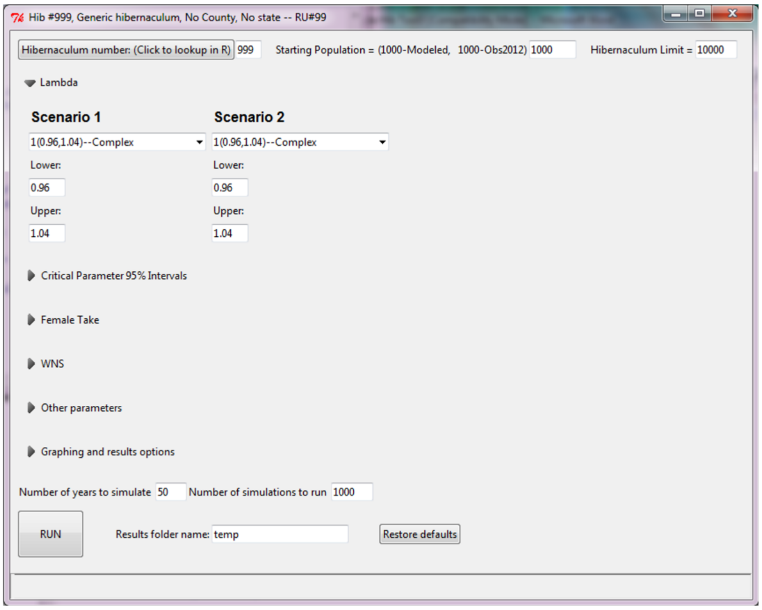

Figure 5 Main GUI page. Clicking on the triangles in the GUI expands the menu for the different subheadings.

```
> library(BatTool)
> example(pop_stochastic)
```

Graphical user interface

The GUI is housed within a demo in the BatTool package. Models from the GUI start with the last year of observed data being year 0 (e.g., if there are observations through 2012 for a hibernacula, year 1 of the output would be 2013).

```
> library(BatTool)
> demo(GUIcode)
```

Running the demo will launch the GUI (Figure 5). Changing the hibernacula number will load data for a new hibernacula after the return key is pressed. Clicking on the "Hibernacula number:" button will launch a table that shows hibernacula information including user-contributed names corresponding to hibernaculum-specific identification numbers. The default starting population is the last population from the last observed year and the default Hibernaculum limit is 1.5× the largest observed population at the hibernacula. Two different scenarios may be run and different options may be set for each scenario. These options are listed under different tabs (Table 3).

The default WNS Year of Infection is based upon the lookup table if the data are available. If the data are not available, the probability of infection for the specified species is used and a random year of infection is used for each simulation. Alternatively, the year of infection may be entered manually; similarly, the probability of infection occurring within a hibernaculum can be adjusted manually.

The default female WNS take parameters for each county are part of the Hibernacula table. These parameters may be changed in either the GUI or the csv file. Example hibernacula 998 contains non-trivial take parameters as an example case. The female take parameters used in the GUI only affect adults. Conversely, the simple model allows either the adult population or juvenile population to suffer take events; similarly, the probability of infection occurring with a hibernaculum can be adjusted manually.

Results from the GUI are stored in a new folder, "ResultsSingleHib\temp". The user may change the **temp**orary folder name within the GUI prior to each simulation; otherwise, past runs will be overwritten. The user can also modify the output figure under the "Graphing and results options" tab. The figure resulting from the GUI (Figure 6) includes the means and credible intervals for two scenarios, any previously observed population data, as well as 4 horizontal lines. The horizontal line at zero represents extinction. The horizontal line at 10,000 bats represents a priority benchmark size for the winter population according to the U.S. Fish and Wildlife Service recovery plan, whereas the horizontal lines at 500 and 2,000 bats represents lower priority hibernacula sizes.

Clicking "RUN" causes the simulations to start and clicking "Restore defaults" reverts settings to their default values. User settings are reported in the results folder.

Table 3 Parameters that may be changed through the GUI

Tab	Parameter name	Parameter table
Lambda	Area of study	Hibernacula table
Lambda	Lower lambda	Hibernacula table
Lambda	Upper lambda	Hibernacula table
Critical parameter Intervals	(All model inputs)	Lambda table
Female take	Take	Hibernacula table
Female take	Begin Year	Hibernacula table
Female take	Duration	Hibernacula table
WNS	Scenario	WN infection table
WNS	Infection Probability	WN infection table
WNS	Year of Infection	WN infection table
Other parameters	Starting Adult proportion	NA
Other parameters	Level of environmental stochasticity	NA
Other parameters	Demographic stochasticity	NA
Graphing and results options	Color	NA
Graphing and results options	Add to existing plot	NA
Graphing and results options	Add (credible intervals)	NA
Graphing and results options	% credible intervals	NA
Graphing and results options	Graph/Results title:	Hibernacula table
NA	Number of years to simulate	NA
NA	Number of simulations to run	NA
NA	Results folder name	NA
NA	Restore defaults	NA
NA	Run	NA

The default values for some parameters are loaded from parameter tables (Table 2). The Critical Parameter tab does not contain any user editable data and is provided as a summary. The take parameters (take, begin year, and duration) may be changed for each season.

Import custom data into the GUI

Custom data may be incorporated into the GUI two different ways. First, values may be directly entered. Second, input tables may be changed. The WNS scenarios may be changed by either changing the default scenario tables or editing the Scenario 1 file (`WNS_other_1.csv`) or Scenario 2 file (`WNS_other_2.csv`) file in the working directory.

Case study

Background

Population viability analysis (PVA) is a quantitative framework for understanding the effects of stressors on populations [6]. This approach allows conservation biologists, decision makers, and risk assessors to compare different management actions (or lack of action). The U.S. Fish and Wildlife Service uses an analytical framework for assessing stressors, which includes PVA as one component. Assessing the effects of wind energy development on the Indiana Bat consists of three steps:

1. Evaluating individual Indiana Bat exposure to action-related stressors and response to that exposure (i.e., likelihood of exposure to wind turbines and the likelihood of death or injury upon exposure);
2. Integrating those individual effects to discern the consequences to the population(s) to which those individuals belong (i.e., what are the effects to the reproductive potential and survival of maternity colonies and hibernacula); and
3. Determining the consequences of any population-level effects to the species at the Recovery Unit and species levels (i.e., will this action affect the likelihood of recovery at these two scales?)

For our case study, we focus on Step 2. Our location is based upon an actual project, but the location has been anonymized for this case study to maintain data confidentiality.

Model settings

We conducted two different assessments. The first was for a maternity colony. The second was for a hibernaculum. All parameters were the same across the two assessments other than the initial population size and hibernaculum limit. A stationary condition ($\lambda \in [0.99 - 1.01]$), but slightly declining population due to model stochasticity, was used. The scenarios used for this assessment did not include White-nose syndrome. Each simulation was run for 50 years and 1,000 simulations were run. The maternity colony assessment had a starting population of 80 and hibernaculum limit of 200. Two bats were taken for 30 years each spring and fall for an annual take of 4 bats per year. This level of take would represent a small, but reasonable loss associated with a wind farm. For the hibernaculum assessment, two different take scenarios were examined. The first scenario only included the loss of 2 bats each spring and fall. This scenario results in the same pattern of take as the maternity colony take scenario. The second hibernaculum scenario included the loss of 300 bats each spring and fall for 30 years for an annual take of 600 bats per year. This level of take would represent take from multiple facilities affecting a hibernaculum. These values are take authorizations requested by wind energy generation concerns. Note that our model does not include spatial structure and this limits the use of our

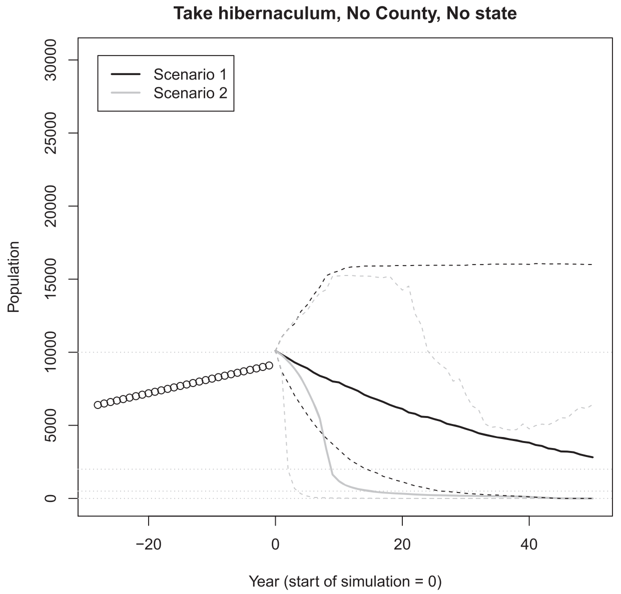

Figure 6 Example GUI output figure. The results from two scenarios are shown. Scenario 2 (in gray) experienced effects of WNS whereas Scenario 1 (in black) did not. The solid lines are the mean outputs and the dashed lines are the 95% credible intervals. Dots are the observed data. The model had the default annual female bats taken for hibernacula 998. The four dashed horizontal lines represent different population thresholds. The line at zero represents extinction. The line at 10,000 bats represents a the highest-priority size for hibernacula from the U.S. Fish and Wildlife Service, the line at 2,000 bats represents the second-highest priority hibernacula size, and the line at 500 bats represents the third group of hibernacula.

model for studying wind energy take at the species level or other large spatial scales. This limitation occurs because the model was developed to initially assess White-nose syndrome at a hibernaculum.

Results and conclusions

The take of 4 females per year (2 during spring, 2 during fall) caused a greater population decline for the maternity colony, but not the hibernaculum (Figure 7, the left panel versus the center panel). The take of 600 females per year was sufficient to increase the rate of decline as well (Figure 7, right panel). Simply evaluating the loss of individuals at hibernaculum or larger scales failed to account for the spatial dynamics of the species. For example, take of only 4 females per year did not yield a detectable effect at the hibernaculum level, but the loss of 4 individuals could lead to the loss of an entire maternity colony if immigration is insufficient to overcome the long-term loss of breeding individuals to take from wind energy development. This impact was not detectable by simply evaluating the loss of 4 individuals from the hibernaculum population because the magnitude of loss relative to the population size was minuscule relative to the stochasticity experienced by the population. These findings indicate

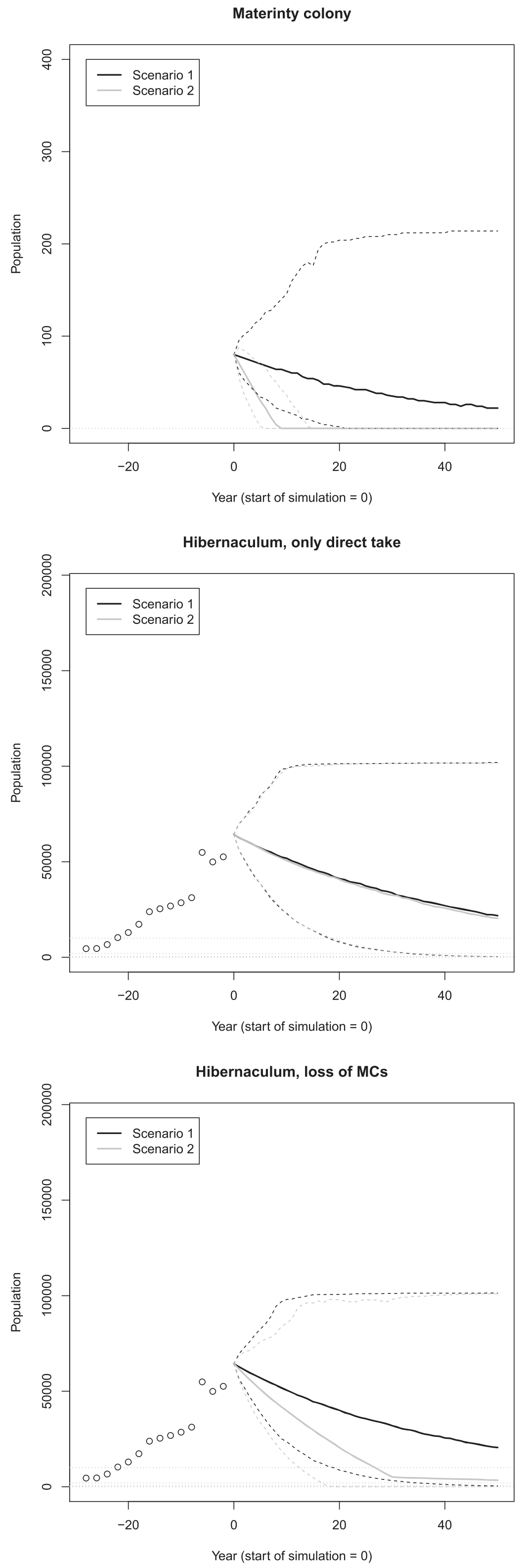

Figure 7 Case study figures. Figures from the case study from three take scenarios. Scenario 2 had take for each set of simulations. The solid lines are the mean outputs and the dashed lines are 95% credible intervals. See the text for differences between scenarios.

that efforts to minimize bat mortality (e.g., altering turbine speeds [12]) may be needed at the development site if real losses are equivalent to those tested in these simulations.

Conclusions

`BatTool` is an **R** package designed to help natural resource managers and decision makers. The package contains a population model accessible through both a GUI and command-line interface. The main functions of the command line are the `main_pop` model function and `pop_stochastic` function. These functions may be used to simulate the population-level effects of WNS and wind energy development. There is also a GUI included as part of this package allowing users who are less comfortable with a command-line interface to use and change the model inputs. Because of the ease of use of the GUI, this package can also be used as part of population ecology or natural resource management courses.

Availability and requirements

This package requires **R** $\geq$ 2.10 and `gWidgetstcltk` $\geq$ 0.0-54. The package is included as part of the online supplemental materials (Additional files 1 and 2).

Additional files

Additional file 1: This file contains the raw BatTool code.

Additional file 2: This file contains the readme file for instillation, the csv files that belong in the working directory, and the complied Windows R package as a ZIP file. This file is all Windows users will need to install and run the package.

Abbreviations

GUI: Graphic user interface; WNS: White-nose syndrome; LBB: Little Brown Bat; Indiana Bat.

Competing interests

The authors declare that they have no competing interests. Any use of trade, product, or firm names are for descriptive purposes only and do not imply endorsement by the U.S. Government. The views expressed in this article are the authors' own and do not necessarily represent the views of the U.S. Fish and Wildlife Service.

Authors' contributions

RAE coded the model as an **R** package and wrote the manuscript. JAS provided technical review of the model, helped test the GUI, and developed the case study, and co-wrote the manuscript. WET oversaw the project, developed the initial model, and co-wrote the manuscript. All authors read and approved the final manuscript.

Acknowledgments

Patrick C. McKann provided early development of the user interface. R. Niver and L. Pruitt provided feedback on tool development that greatly enhanced this tool's ease of use.

Author details

[1]Upper Midwest Environmental Science Center, U.S. Geological Survey, 2630 Fanta Reed Road, 54603 La Crosse, WI, USA. [2]Cherokee Nation Businesses, 165 South Union Blvd Suite 700, 80228 Lakewood, CO, USA. [3]Division of Endangered Species, U.S. Fish and Wildlife Service, 555 Lester Avenue, 54650 Onalaksa, WI, USA.

Received: 08 March 2014 Accepted: 11 April 2014
Published: 6 May 2014

References

1. Frick WF, Pollock JF, Hicks AC, Langwig KE, Reynolds DS, Turner GG, Butchkoski CM, Kunz TH: **An emerging disease causes regional population collapse of a common north american bat species.** *Science* 2010, **329**(5992):679–682.
2. Froschauer A, Racey M, Riley C: **Review finds engangered species protection may be warranted for two bat species.** Technical report, U.S. Fish and Wildlife Service. 2011.
3. 32FR4001: **Notices.** *Fed Regist* 1967, **32:**4001.
4. Kunz TH, Braun de Torrez, E, Bauer D, Lobova T, Fleming TH: **Ecosystem services provided by bats.** *Ann NY Acad Sci* 2011, **1223:**1–38.
5. Altringham JD: *Bats: From Evolution to Conservation*. Oxford: Oxford University Press; 2011.
6. Morris WF, Doak DF: *Quantitative Conservation Biology*. Sunderland: Sinauer Associates; 2002.
7. Caswell H: *Matrix Populations Models*. Sunderland: Sinauer Associates Inc.; 2001.
8. Thogmartin WE, Sanders-Reed CA, Szymanski JA, McKann PC, Pruitt L, King RA, Runge MC, Russell RE: **White-nose syndrome is likely to extirpate the endangered Indiana bat over a large part of its range.** *Biol Conserv* 2013, **160:**162–172.
9. R Core Team: *R: A Language and Environment for Statistical Computing*. Vienna: R Foundation for Statistical Computing; 2013.
10. Gotelli NJ: *A Primer of Ecology*. 4th edition. Sunderland: Sinauer; 2008.
11. Allen LJS: *Introduction to Mathematical Biology*. Upper Saddle River: Pearson/Prentice Hall; 2007.
12. Arnett EB, Huso MM, Schirmacher MR, Hayes JP: **Altering turbine speed reduces bat mortality at wind-energy facilities.** *Front Ecol Environ* 2010, **9**(4):209–214.

doi:10.1186/1751-0473-9-9
Cite this article as: Erickson *et al.*: BatTool: an R package with GUI for assessing the effect of White-nose syndrome and other take events on *Myotis* spp. of bats. *Source Code for Biology and Medicine* 2014 **9**:9.

Submit your next manuscript to BioMed Central and take full advantage of:

- **Convenient online submission**
- **Thorough peer review**
- **No space constraints or color figure charges**
- **Immediate publication on acceptance**
- **Inclusion in PubMed, CAS, Scopus and Google Scholar**
- **Research which is freely available for redistribution**

Submit your manuscript at
www.biomedcentral.com/submit

BioMed Central
